# A question is “what are the optimal targets for anticoagulant therapies?”

**DOI:** 10.1186/s40560-020-0434-9

**Published:** 2020-02-14

**Authors:** Nobuyuki Yokoyama, Shunsuke Takaki, Masashi Yokose, Kaori Kuwabara, Akiko Anzai, Takako Hamada, Shizuka Kashiwagi, Kenta Okamura, Yoh Sugawara, Takahisa Goto

**Affiliations:** Department of Anesthesiology and Critical Care Medicine, Yokohama City University Hospital, 3-9 Fukuura Kanazawaku, Yokohama, Kanagawa 236-0004 Japan

**Keywords:** Disseminated intravascular coagulation (DIC), Antithrombin, Recombinant thrombomodulin

## Abstract

A high mortality rate is found among septic patients with disseminated intravascular coagulation (DIC). Anticoagulants have been used for treating septic DIC especially in Japanese clinical settings; however, their effectiveness is quite controversial across studies. According to several randomized controlled trials and meta-analyses, antithrombin and recombinant thrombomodulin had no therapeutic benefit in the treatment of sepsis. However, the majority of the previous research did not discuss “septic DIC” but simply “sepsis”, and some reviews showed that anticoagulants were benefit only in septic DIC. Although immunothrombosis plays an important role in early host defense, it can lead to DIC and organ failure if dysregulated. Therefore, we advocate anticoagulant therapies might have beneficial effects, but research on optimal patient selection is currently lacking.

## Background

Sepsis is a very serious disease with approximately 35% of septic patients meeting the diagnostic criteria for DIC [1]. The Japanese sepsis registry revealed that the 28-day mortality rate and the hospital mortality rate for DIC patients were higher than those for non-DIC patients [2]. However, an unresolved clinical question is whether a specific treatment for septic DIC reduces mortality. In this review, we discuss anticoagulation therapy for those with septic DIC and its effectiveness.

## Main body

### Consistency of evidence

Antithrombin (AT) and recombinant thrombomodulin (rTM) are widely used in anticoagulant therapy for patients with septic DIC in Japanese clinical settings. The Surviving Sepsis Campaign Guideline (SSCG 2016) recommends that AT should not be used for septic patients (strong recommendation); these guidelines make no statements regarding rTM [3]. Nevertheless, this recommendation is intended for sepsis, but not for septic DIC. In the Japanese guideline for sepsis, AT is recommended for septic DIC patients with low AT activity [4]. This recommendation is based on a study that investigated patients with septic DIC [5].

In the large Kybersept RCT, AT did not improve survival prognosis but rather significantly increased bleeding complications [6]. However, this trial recruited patients with severe sepsis, including those with and without septic DIC. As a result, only 40.7% of patients (229 of 563) had DIC in the trial. This means that the conclusion may not be applicable to septic DIC patients. Similarly, a meta-analysis that evaluated a cohort from the Kybersept trial in 2016 did not demonstrate the effectiveness of AT [7]. On the contrary, a post-hoc analysis of this trial investigating septic DIC patients showed that AT improved survival prognosis profoundly [5]. More recently, Wiedermann performed a meta-analysis of its use in patients with sepsis and DIC and reported a beneficial effect of antithrombin on the mortality [8].

In regard to rTM, a phase 2b multinational randomized trial showed a better 28-day mortality [9], but a phase 3 SCARLET trial stated that it did not improve survival prognosis [10]. One reason is, similar to the trial on AT use, this trial included sepsis patients with abnormal coagulation, even though they did not fulfill the DIC criteria. Another reason is concomitant uses of heparin that counter a benefit of rTM. Although a recent meta-analysis including SCARLET trial found trend toward improved survival, this effect was not statistically significant [11]. Therefore, further rTM trials for septic DIC patients are desirable.

Two reviews made an attempt to reveal the effectiveness of anticoagulant therapy for those with septic DIC. In one review, Japanese septic patients were divided into three groups; group 1 consisted of those with normal coagulation, group 2 with abnormal coagulation (i.e., patients with a single coagulation-related parameter change), and group 3 consisted of those with DIC (i.e., patients who fulfilled any of the DIC diagnostic criteria). This particular review pointed out that the risk ratio for death was significantly reduced in group 3, but in groups 1 and 2, anticoagulant therapy was effective only in patients with septic DIC [12]. The other review summarized the septic DIC studies conducted in Japan. It presented survival curves according to patient profiles when anticoagulant therapy was applied. Improvement was observed only in the survival curve of patients with septic DIC or SOFA scores of 13–17 [13]. From these reviews, anticoagulants may be effective only for septic DIC, not for sepsis without DIC. To confirm this opinion, large prospective trials for anticoagulants are needed.

### Immunothrombosis

Immunothrombosis is an immune response in which blood clots are formed to isolate bacterial infections [14]. It has several protective mechanisms, such as compartmentalizing pathogens inside the blood clots (thrombosis), working as a barricade to prevent their invasion and spreading, mobilizing immunological factors to kill pathogens, and activating leucocytes with fibrinogen and fibrin. When occurring locally, it is beneficial for the body. However, once getting beyond control, immunothrombosis contributes to aberrant thrombus formation and thrombotic diseases including DIC [15]. From this point of view, anticoagulant therapy may be necessary for those with septic DIC to modulate thrombotic reactions.

Furthermore, besides their anticoagulant effects, anticoagulant agents play an important role in immunomodulating and protecting vascular endothelium effects. For example, AT is effective in protecting the glycocalyx [16] because glycocalyx degeneration is known to be associated with the increased severity of sepsis [17].

### Optimal targets of treatment for septic DIC

Septic DIC is the overexpression of the host defense system as discussed above, and it can cause organ failure. Evidences also showed that anticoagulants may be effective only for septic DIC patients. Therefore, we should use anticoagulants for septic DIC, not for sepsis. DIC is often diagnosed using the Japanese Association for Acute Medicine (JAAM) DIC scoring system. However, the JAAM scoring system has a high sensitivity, but a low specificity. Therefore, this raises the question: How do we choose the best candidates for anticoagulant therapy? According to a recent article, Iba et al. stated that anticoagulant therapy was an important consideration in septic DIC and early and rapid diagnosis was important for this therapy [18]. In the other article, those with “sepsis,” “DIC,” and “high disease severity” might be optimal patients for anticoagulant therapy [19]. The authors of this article used SOFA or APACHE II scores to determine disease severity. This may be reasonable because these score reflect organ failure directly. However, without enough evidence, the authors recommended the need for new directions for future clinical research.

## Conclusion

Research findings regarding anticoagulation methods and the effects of anticoagulants on vascular endothelium in those with dysregulated immunothrombosis and other research specifically focusing on septic DIC indicated the effectiveness of anticoagulant therapy for sepsis management. Therefore, future research should address the following question: “How do we apply this therapy for optimal targets?”

## Data Availability

Data sharing not applicable to this article as no datasets were generated or analyzed during the current study.

## Abbreviations

AT: Antithrombin
DIC: Disseminated intravascular coagulation
JAAM: Japanese Association for Acute Medicine
rTM: Recombinant thrombomodulin
